# Moxibustion with Walnut Shell Spectacles Could Improve the Objective Symptoms and Tear Film Stability of Patients with Dry Eye Disease: A Randomized Controlled Trial

**DOI:** 10.1155/2022/1773444

**Published:** 2022-11-30

**Authors:** Guoliang Zhang, Weiwei Fu, Jing Xu, Pei Hu, Yi Zhang, Zijin Sang, Wenting Wu, Kun Zheng, Lie Wu, Zhishun Liu

**Affiliations:** ^1^Department of Ophthalmology, Guang'anmen Hospital, China Academy of Chinese Medical Sciences, Beijing, China; ^2^Department of Acupuncture, Guang'anmen Hospital, China Academy of Chinese Medical Sciences, Beijing, China; ^3^Department of Ophthalmology, Guangdong Province Hospital of Traditional Chinese Medical, Guangzhou, China

## Abstract

**Objectives:**

This study aims to evaluate the efficacy and acceptability of moxibustion with walnut shell spectacles in treating dry eye disease (DED) patients and to provide treatment options.

**Methods:**

126 DED patients were randomly allocated into the moxibustion group (treated by moxibustion with walnut shell spectacles, 64 cases) and the artificial tears group (treated with sodium hyaluronate eye drops, 62 cases). Evaluate the changes in the ocular surface disease index (OSDI), the visual analogue scale (VAS) of ocular discomfort, the tear film break-up time (TBUT), corneal fluorescein staining (CFS), and the Schirmer I test during the trial at baseline and after 1-week, 2-week, 3-week, and 4-week treatment. Evaluate the OSDI scale and the ocular symptom VAS scale one month after the end of treatment.

**Results:**

There were no significant differences in baseline characteristics between the two groups. For OSDI scores, the results showed that the efficacy of the moxibustion group was no less than that of the artificial tear group. For VAS of ocular discomfort, both groups significantly reduced their score compared with baseline, and for the moxibustion group, the decrease was more significant. For TBUT, FAS, and PPS, results showed that the efficacy of the moxibustion group was significant in both eyes after 4 weeks of treatment, but the right eye was in the artificial tear group. For CFS and Schirmer I test scores, there was no significant effect for both groups.

**Conclusion:**

Moxibustion with walnut shell spectacles could improve the clinical symptoms and tear film stability of DED patients; however, it has no significant efficacy on improving corneal injury and tear secretion, just the same as sodium hyaluronate eye drops. Nevertheless, moxibustion with walnut shell spectacles may have better effects on the self-assessment of ocular discomfort than sodium hyaluronate eye drops.

## 1. Introduction

Dry eye disease (DED, also known as dry eye syndrome) is a common ocular condition, referring to a group of disorders of the tears and ocular surface, such as ocular irritation, redness, mucus discharge, alternating vision, and decreased tear meniscus or plugged meibomian glands, which are caused by reduced tear production or tear film instability [1, 2]. Many factors could lead to DED. While elders and female gender have been identified as major risk factors [3, 4]. Using visual display terminals [5], taking glaucoma medication containing benzalkonium chloride [6] or anticholinergic drugs (e.g., antidepressant, antipsychotic, anti-Parkinson's disease, and antihistamine drugs) [7], some immune diseases (e.g., rheumatoid arthritis [8], Sjogren's syndrome [9]), use of contact lenses [10], vitamin A deficiency [11], and smoking use were also associated with an increased risk of DED [3]. DED has a substantial impact on the life quality of afflicted individuals. Also, it may compromise the results of corneal, cataract, and refractive surgery [1]. DED can lead to dysfunction of the tear film, lacrimal gland system, eyelid, conjunctiva, and cornea [12], dramatically affect the life quality of patients, lead to reduced effectiveness at work [13–15], and impose a substantial economic burden on the patients and society [13]. In China, the incidence rate of DED has reached 21%–30%, and DED patients have accounted for more than 30% of the total ophthalmology outpatients [16], with an estimated prevalence of 5% to 35% worldwide [17]. According to its risk factors, we believe that the incidence of DED would increase, since the current widespread use of electronics and the advent of social aging.

Ocular lubricants were recommended as the first step for treating DED [18]. Also, artificial tears are often effective in relieving symptoms of DED by replenishing deficient tear volume. Sodium hyaluronate eye drops are commonly used in clinics as sodium hyaluronate can hold large quantities of water, thus lubricating surrounding structures as well as improving tear film stability and promoting epithelial wound healing [19, 20], while tear substitutes are usually used frequently and chronically [21].

Moxibustion with walnut shell spectacles is a characteristic therapy of Guang'anmen Hospital, developed on the basis of walnut shell moxibustion, and mainly composed of an eye moxibustion frame, a walnut shell soaked with wolfberry and chrysanthemum liquid, and moxibustion strips. Moxibustion with a walnut shell was first recorded by Shicheng Gu for treating surgical ulcers in the Qing dynasty. Then, moxibustion with walnut shell spectacles was reformed by us, combining Shicheng Gu's experience with our clinical practice, and is mainly used for the treatment of optic nerve atrophy and myopia. In recent years, our clinical practice found that moxibustion with walnut shell spectacles could also improve the symptoms of DED [22]. Therefore, we conducted a randomized controlled trial to evaluate the efficacy and safety of moxibustion with walnut shell spectacles on DED and provided treatment options for the treatment of DED.

## 2. Materials and Methods

We conducted a randomized controlled trial with artificial tears as a control to obtain high-quality evidence-based evidence on the efficacy and safety of moxibustion with walnut shell spectacles in the treatment of DED. This study protocol was reviewed and approved by the Ethics Committee of Guang'anmen Hospital, China Academy of Chinese Medical Sciences (NO.2016-121-KY), and was registered in the Clinical Trials (NCT03116776).

### 2.1. Participants

All cases were included from the Department of Ophthalmology and Acupuncture of Guang'anmen Hospital, China Academy of Chinese Medical Sciences, from March 2017 to May 2018. A total of 130 patients were included.

Diagnostic criteria are as follows: refer to the diagnostic criteria of the consensus of clinical experts on the dry eye (2013) [23].

(1) DED symptoms (dry eyes, foreign body sensation, burning sensation, soreness, visual fatigue, photophobia, etc.). (2) Tear film break-up time (BUT) <5 s or Schimer I test without anaesthesia <5 mm/5 min. (3) 5 s< BUT<10 s or 5 mm/5 min <Schimer I test without anaesthesia <10 mm/5 min; and the corneal fluorescein staining test was positive.

If patients met (1) (2) or (1) (3) at the same time, the diagnosis could be made.

Inclusion criteria are as follows:

(1) Meet the diagnostic criteria for DED. (2) Between the ages of 18 and 75. (3) Sign the informed consent and volunteer to participate in this test.

Exclusion criteria are as follows:

(1) Patients with Sjögren's syndrome. (2) Patients suffering from mental disorders, severe heart, liver, or kidney disease, hematopoietic system diseases, or malnutrition. (3) A history of surgical operations related to the eye in the last 6 months. (4) Changes in ocular structure, such as conjunctival scarring, corneal ulcer, atresia at the lacrimal opening, complete atrophy of the accessory lacrimal gland, and eyelid deformity. (5) Complicated with other eye diseases, such as glaucoma, conjunctivitis, uveitis, fundus hemorrhage, and optic nerve diseases. (6) Pregnant or nursing women. (7) Or those who are allergic to moxibustion or related drugs used in this study (sodium hyaluronate eye drops, medlar, and chrysanthemum). (8) Treated with other dry eye medications in the past 2 weeks. (9) Treated with moxibustion with walnut shell spectacles in the past 30 days. Those who met any of the above criteria were excluded.

### 2.2. Groups and Interventions

#### 2.2.1. Moxibustion Group: Treated by Moxibustion with Walnut Shell Spectacles

**Figure 1:**
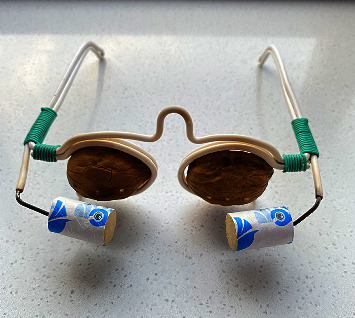
Walnut shell spectacles.

Walnut shell spectacles were made by us. The frame is made of wire. While preparing the walnut shell, choose one dry walnut, cut the walnut in the middle line, remove the walnut kernel, and take the shell for use (the shell should be crackless); place 10 grams each of medlar and chrysanthemum in water, boil the water, and immerse the walnut shell in the liquid for about 30 min to allow the liquid to penetrate the walnut shell (the walnut shell could be reused). (Figure 1).

**Figure 2:**
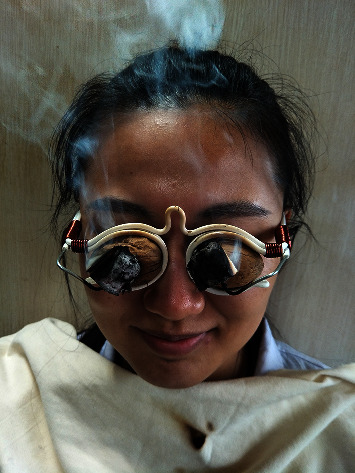
Patient with moxibustion.

Moxibustion steps: let the patients sit or lie down; close their eyes (remove glasses or corneal contact lenses); place a white cloth in front of the patient's chest before moxibustion if the patient was sitting, to avoid falling off the moxa ash; take out the soaked walnut shell, fill it with the appropriate amount of medlar and chrysanthemum, and fix the walnut shell on the frame; hitch one section of moxa (diameter: 2.0 cm, length: 1.5 cm) on each wire in front of the spectacle frame; ignited the moxa section from the inside; let the patients put on the spectacles to do moxibustion (Figure 2).

The course of treatment: moxibustion every other day; used three moxa sections on each side (about 45 min); 3 times a week; treated for 4 weeks.

#### 2.2.2. Artificial Tears Group: Treated by Sodium Hyaluronate Eye Drops

Applied one drop of sodium hyaluronate eye drops (produced by Cantian Pharmaceutical Co., Ltd., batch number H20130583) each time for each eye, 4 times a day; treated for 4 weeks.

#### 2.2.3. Sample Size Calculation

The main outcome index of this trial is the change in ocular surface disease index (OSDI) score from baseline compared with that after 4-week treatment. According to relevant clinical studies [24], comparing the OSDI score at baseline with that after 4-week treatment, the difference value of patients treated by moxibustion with walnut shell spectacles was 33.4 ± 25.4, while that of patients treated with sodium hyaluronate eye drops was 21.7 ± 16.1. According to the different test formulas of the two groups, it is given as follows:

(1) $$\begin{array}{c} n=\frac{\left(U\alpha +U\beta \right)\times \left(1+1/k\right)s^{2}}{\delta^{2}}\text{,} \end{array}$$

*k* = 1 (1 : 1 ratio for 2 groups).

Taking *α* = 0.05 and *β* = 0.2, then *n* = 53, considering a rate of 20% drop, the calculated required sample size for each group was 64 cases.

#### 2.2.4. Randomization Program

The randomization program was compiled by the Office of the National Good Clinical Practice of Guang'anmen Hospital. Random numbers with block randomization were generated by using the SAS software (SAS Version 9.4, SAS institute. Inc, Cary, NC, USA), setting the size length to 4, and setting the A group to be treated by moxibustion with walnut shell spectacles while the B group was treated by sodium hyaluronate eye drops. Then, hide the groups with opaque sealed envelopes containing the random code. Enable envelopes sequentially to determine grouping according to the order of the included patient's participation.

### 2.3. Outcomes

The outcome assessment included objective questionnaires and subjective ophthalmologic tests.

#### 2.3.1. Ocular Surface Disease Index (OSDI)

The OSDI changes between the two groups were the primary outcomes. OSDI is a validated and reliable questionnaire that could provide a rapid assessment of the symptoms of ocular irritation consistent with dry eye disease and their impact on vision-related functioning. It consists of 12 items, as 6 questions are related to vision, 3 questions are about ocular symptoms, and 3 questions are referred to environmental triggers. Each question has a score of zero to four, while scores regarding the frequency of DED symptoms are “never,” “sometimes,” “often” and “constantly”; while scores about the degree of DED symptoms can be reported as “none,” “some,” “intense” and “very intense.” The OSDI score was calculated according to the following formula: OSDI = ((sum of scores of all questions answered) *∗* 100)/((total number of questions answered) *∗* 4). The score ranges from 0 to 100, and the higher the score one got, the more severe the dry eye was. Each time, evaluate the average dry eye condition of the past week. A version translated into Chinese was used [25, 26].

#### 2.3.2. Visual Analogue Scale (VAS) of Ocular Discomfort

A 100-millimeter VAS was used for the self-assessment of ocular discomfort by participants. Ocular symptoms related to dry eye (e.g., ocular itching, a foreign body sensation, burning sensation, pain and dryness, blurred vision, a sensation of photophobia, ocular redness, and sensations of tearing) were quantified and summarized on a standard 100-millimeter VAS scale. 0 indicates “no eye discomfort” and 100 indicates “the most intolerable eye discomfort.” The patient marked a point on the analog scale that best represented the degree of discomfort; then, the distance from 0 mm to the point the patient marked was the VAS score. Each time, let the participant assess the average ocular discomfort of the last 24 hours.

#### 2.3.3. Tear Film Break-Up Time (TBUT)

Tear Film BUT is a test for assessing tear film stability, which should be tested before other operations that could disturb the ocular surface. To test TBUT, a drop of sodium fluorescein (1%) was administered in the inferior cul de sac of the eye by pulling down on the lower lid and gently touching the bulbar conjunctiva with a glass rod, and participants were requested to blink a few times to ensure adequate mixing of the fluorescein dye with tears. With the regular room lights turned off, the ophthalmologist shined a cobalt blue light in a slit-lamp on the eye, checked the tear film by a wide band of cobalt blue light, and measured the time interval between the last complete blink and the appearance of the first corneal dark spot by a stopwatch, and the mean of 3 measurements was regarded as the TBUT value. A value below 10 indicates that the lipid layer of the tear film is compromised [27].

#### 2.3.4. Corneal Fluorescein Staining (CFS)

Corneal fluorescein staining (CFS) is an important clinical tool for diagnosing corneal injury or assessing the viability of the epithelium [28]. The ophthalmologist placed a drop of 2% sodium fluorescein in the bulbar conjunctiva of the lower lid with a glass rod. Let the patient blink to distribute the dye. Then, cobalt blue light was used to observe whether the corneal epithelium of the patient was stained. If it were stained yellow and green, the integrity of the corneal epithelial cells would be destroyed. Scored using a 12-point method: the cornea was divided into 4 quadrants; each quadrant was 0–3 points; no staining was 0 points; staining 1–30 points was 1 point; more than 30 points of staining but no staining fusion was 2 points; corneal point staining fusion, filamentous, and the ulcer were 3 points [20].

#### 2.3.5. Schirmer I Test (Without Anaesthesia)

The Schirmer I test is the most commonly used diagnostic method to measure the basic quantity of tear secretion [29]. Placed Schirmer test paper (Color Bar, Eagle Vision, USA) in the conjunctival sac at the 1/3 junction of the lower eyelid, asked the patient to close his eyes or look down, took out the test paper after 5 minutes, and measured the wet length from the bend. The normal value was 10–15 mm [20].

#### 2.3.6. Evaluation Time Points

OSDI, VAS, TBUT, CFS, and Sch I were all evaluated at baseline and changed after 1-week, 2-week, 3-week, and 4-week of treatment. Follow-up: evaluate the OSDI scale and the ocular symptom VAS scale one month after the end of treatment. All patients were treated for both eyes, and the results of a binocular ophthalmic examination were collected for data statistics.

### 2.4. Statistical Methods

Statistical analyses were conducted by using the statistical package for the social sciences (SPSS) version 22.0 (SPSS Inc., Chicago, IL, USA). All data were expressed as the mean ± SEM. If the data distribution conformed to normal, the comparison between groups was tested by two independent samples *t*, and the comparison before and after the treatment in the group was tested by paired samples *t*. If the data did not conform to a normal distribution, it was expressed in the median and interquartile spacing M (qr) using a nonparametric test. A value of *P* < 0.05 was considered statistically significant. The Full Analysis Set (FAS) refers to all randomized patients. The patients who completed acupuncture treatment and follow-up according to the protocol did not deviate from the important protocol and completed the evaluation of the main outcomes constituted the Per-Protocol Set (PPS) of this study.

## 3. Results

**Figure 3:**
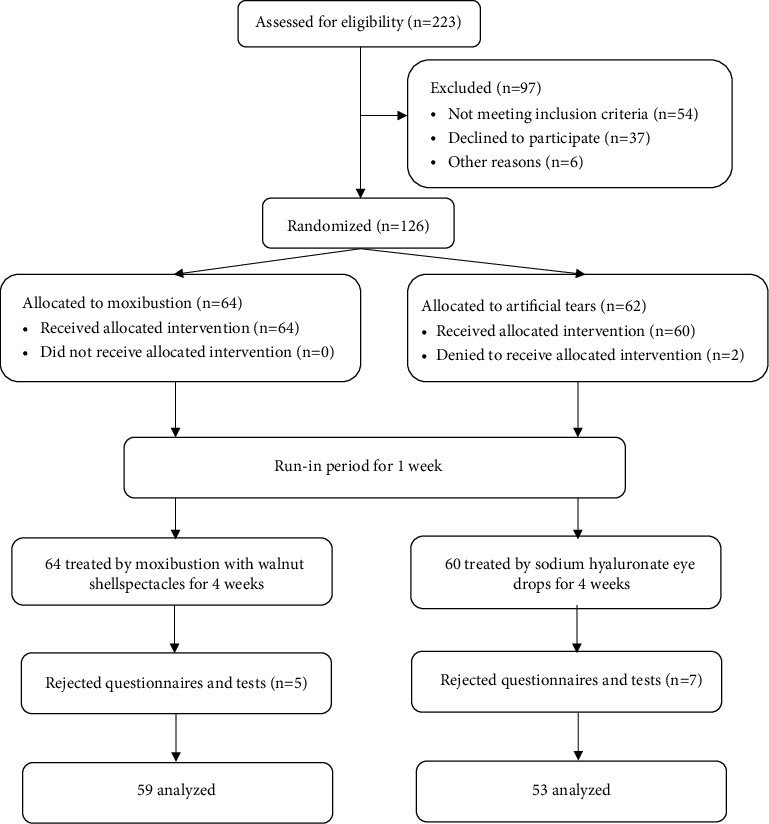
Flow diagram.

A total of 126 DED patients were included in the grouping process. There were 64 cases allocated to the moxibustion group and 62 cases to the artificial tear group. 2 participants in the artificial tear group dropped out before treatment. 5 patients in the moxibustion group rejected questionnaires and tests during the treatment, while 7 participants in the artificial tear group rejected them (Figure 3).

### 3.1. Baseline Characteristics

Demographic data and clinical characteristics of DED participants in two groups are presented in Table 1. There were no significant differences in baseline characteristics between the moxibustion and artificial tear groups.

### 3.2. Outcomes

#### 3.2.1. Ocular Surface Disease Index (OSDI)

Changes in OSDI scores of two groups during the trial are presented in Table 2. There is no statistical significance in OSDI scores between moxibustion and artificial tear groups at baseline for both FAS (*P*=0.388) and PPS analysis (*P*=0.123). After 1 month of end treatment, there is no statistical difference between groups for the OSDI scores.

For FAS analysis, OSDI scores are significantly reduced in the moxibustion group after 1- (*P*=0.035), 2- (*P* < 0.001), 3- (*P* < 0.001), and 4-weeks (*P* < 0.001) of treatment, and 2- (*P*=0.009), 3- (*P* < 0.001), and 4-weeks (*P*=0.001) in the artificial tear group, and there is no statistical difference between groups. After 4-weeks of end treatment, there is no statistical difference between groups for the OSDI scores.

For PPS analysis, OSDI scores are significantly reduced in the moxibustion group after 1- (*P*=0.027), 2- (*P* < 0.001), 3- (*P* < 0.001), and 4-weeks (*P* < 0.001) of treatment, and 2- (*P*=0.006), 3- (*P* < 0.001), and 4-weeks (*P*=0.003) in the artificial tear group, and there is no statistical difference between groups. After 1 month of end treatment, there is no statistical difference between groups for the OSDI scores.

#### 3.2.2. Visual Analogue Scale (VAS) of Ocular Discomfort

Changes in VAS scores between the two groups during the trial are presented in Table 3. At a baseline, there is no statistical significance in VAS scores between moxibustion and artificial tear groups for both FAS (*P*=0.672) and PPS analysis (*P*=0.197).

For FAS, compared with baseline, VAS scores of two groups are both significantly reduced after 1- (*P* < 0.001), 2- (*P* < 0.001), 3- (*P* < 0.001), and 4-week (*P* < 0.001) of treatment in the moxibustion group, and 1- (*P* < 0.001), 2- (*P* < 0.001), 3- (*P* < 0.001), and 4-week (*P* < 0.001) of treatment in the artificial tear group. Compared with the artificial tear group, VAS scores of the moxibustion group significantly reduced after 2- (*P*=0.017), 3- (*P*=0.037), and 4-week (*P*=0.003) of treatment. After 1 month of end treatment, there is no statistical difference between groups for the VAS scores.

For PPS, compared with baseline, VAS scores of two groups are both significantly reduced after 1- (*P* < 0.001), 2- (*P* < 0.001), 3- (*P* < 0.001), and 4-week (*P* < 0.001) of treatment in the moxibustion group, and 1- (*P*=0.001), 2- (*P* < 0.001), 3- (*P* < 0.001), and 4-week (*P*<0.001) of treatment in the artificial tear group. Compared with the artificial tear group, VAS scores of the moxibustion group significantly reduced after 2- (*P*=0.020) and 4-week (*P*=0.015) of treatment. After 1 month of end treatment, there is no statistical difference between groups for the VAS scores.

#### 3.2.3. Tear Film Break-Up Time (TBUT)

Changes in TBUT between the two groups during the trial are presented in Table 4. There is no statistical significance in TBUT between moxibustion and artificial tear groups at baseline for both FAS (left eye: *P*=0.137; right eye: *P*=0.421) and PPS analysis (left eye: *P*=0.179; right eye: *P*=0.484).

For FAS, compared with baseline, TBUT is significantly reduced after 4-week treatment in the moxibustion group for both the left eye (*P* < 0.001) and for the right eye (*P*=0.001), and right eye (*P*=0.002) in the artificial tear group. Also, there is no statistical difference between groups for the TBUT.

For PPS, compared with baseline, TBUT is significantly reduced after 4-week treatment in the moxibustion group for both the left eye (*P*=0.001) and for the right eye (*P*=0.003), and right eye (*P*=0.010) in the artificial tear group. Also, there is no statistical difference between groups for the TBUT.

#### 3.2.4. Corneal Fluorescein Staining (CFS)

Changes in CFS scores between the two groups during the trial are presented in Table 5. There is no statistical significance in CFS between moxibustion and artificial tear groups at baseline for both FAS (left eye: *P*=0.774; right eye: *P*=0.390) and PPS analyses (left eye: *P*=0.356; right eye: *P*=0.136).

For FAS, compared with baseline, CFS is significantly reduced after 2-week treatment in the artificial tear group for the left eye (*P*=0.004), and there is a statistical difference between groups for the CFS (*P*=0.027).

For PPS, compared with baseline, CFS is significantly reduced after 2-week treatment in the artificial tear group for the left eye (*P*=0.001), and there is a statistical difference between groups for the CFS (*P*=0.013).

#### 3.2.5. Schirmer I Test

Changes of Schirmer I test scores of two groups during the trial are presented in Table 6. There is no statistical significance in Schirmer I test scores between moxibustion and artificial tear groups at baseline for both FAS (left eye: *P*=0.234; right eye: *P*=0.588) and PPS analysis (left eye: *P*=0.137; right eye: *P*=0.895). There is statistical significance in the Schirmer I test scores of the right eye in the artificial tear group compared to baseline for both FAS (*P*=0.028) and PPS analysis (*P*=0.019), but not for the moxibustion group.

## 4. Discussion

As mentioned in the introduction, DED is a common ophthalmologic disorder that has a substantial impact on the life quality of afflicted individuals owing to discomfort and visual disability. Both extrinsic and intrinsic factors may contribute to DED. Also, intrinsic factors including autoimmune responses, hormonal disturbances, inherited disorders, and dysbiosis of gut microbiota [30–35]. Extrinsic factors include environmental effects, smoking, and ophthalmic surgical procedures [36, 37]. Currently, the most important method for treating DED is using artificial tears locally to improve ocular surface humidity and lubrication capacity [38, 39]. Some topical drugs are approved for treating DED with anti-inflammatory and immunomodulatory effects, such as Cyclosporin A [40], corticosteroids [41], as well as emerging therapeutics such as lacrimal and diquafosol sodium [42–46]. Also, there are still challenges in the treatment of DED [47].

This study shows that moxibustion is similar to artificial ears in improving eye symptoms. In this trial, for OSDI scores, the results show that the efficacy of the moxibustion group was no less than that of the artificial tear group. For VAS of ocular discomfort, both groups significantly reduced their scores compared with baseline, and for the moxibustion group, the decrease was more significant. For TBUT, FAS and PPS results showed that the efficacy of the moxibustion group was significant in both eyes after 4 weeks of treatment, but only in the right eye in the artificial tear group. For CFS and Schirmer I test scores, there is no significant effect for both groups. Combined with previous studies, we also found no adverse events from moxibustion, which can reduce the side effects caused by local medication, which also shows the safety of the clinical application of moxibustion. At the same time, patients are treated with moxibustion, which has better effects on the self-assessment of ocular discomfort than sodium hydroxynate eye drops, which is considered the advantage of moxibustion.

Moxibustion, acting as an important part of traditional Chinese medicine (TCM) and natural therapy, has a wide range of indications and has a positive effect on many chronic and severe diseases in Chinese clinics. Because of the particularity of eye tissue, we created moxibustion with walnut shell spectacles. In our clinical practice, it is mainly used for treating optic nerve atrophy, or myopia. In recent years, we have found it can also improve the symptoms of dry eye. So, we designed this trial to evaluate its efficacy.

Impairment of both innate and adapted immunity may lead to DED [48, 49]. Existing studies suggest that a large number of lymphocyte infiltration can be seen in the lacrimal gland and conjunctiva of DED patients, and inflammatory cells cause proinflammatory cytokine production [50], such as interleukin 1*β* (IL-1*β*), tumor necrosis factor *α* (TNF-*α*), and interleukin 6 (IL-6) [51] TCM theory holds that moxibustion has the functions of warming and dredging channels and collaterals, regulating qi and blood [52]. Also, studies suggest that moxibustion has an anti-inflammatory and immune-regulation effect [53]. The energy produced by the radiation process of moxibustion may provide activation for the pathological cells lacking energy and then regulate the body's immune function [54]. Therefore, we speculate that the potential mechanism of moxibustion in the treatment of DED is related to the inhibition of ocular inflammation and the regulation of immunity.

We conducted a randomized controlled trial that showed that moxibustion with walnut shell spectacles could effectively treat DED and that the effect was comparable to that of sodium hyaluronate eye drops, followed by moxibustion with walnut shell spectacles, could improve the clinical symptoms and tear film stability of patients with DED. But considering the number of patients included in this trial, subgroup analysis has not been carried out on disease type (water deficiency dry eye and hyper-evaporation dry eye), gender, or age so as to determine the best applicable population of moxibustion with walnut shell spectacles. At the same time, considering the treatment mode of the study, the blind method has not been implemented in this study, and there may be some bias in the evaluation of the outcome.

In conclusion, moxibustion with walnut shell spectacles could improve the clinical symptoms and tear film stability of patients with DED, and the effect is comparable to that of sodium hyaluronate eye drops. Therefore, patients with DED who like moxibustion can choose moxibustion with walnut shell spectacles as the preferred treatment. Meanwhile, it's much better to use methods that could prompt tear secretion and corneal epithelium regeneration for DED patients.

## Figures and Tables

**Table 1:** Baseline characteristics.

Characteristics	FAS analysis			PPS analysis		
	Moxibustion group (*n* = 59)	Artificial tear group (*n* = 53)	*P* value	Moxibustion group (*n* = 64)	Artificial tear group (*n* = 60)	*P* value
Gender			0.064			0.101
Male (*n*)	12	20	—	11	17	—
Female (*n*)	52	40	—	48	36	—

Age	44.44 ± 16.12	43.15 ± 15.13	0.648	45.31 ± 16.01	42.19 ± 15.28	0.296
Symptom duration	3.69 ± 5.72	3.16 ± 3.22	0.821	3.82 ± 5.92	3.05 ± 3.24	0.879
Operation	8	5	0.449	7	4	0.443
Long-period wearing contact lens	6	4	0.580	4	4	0.875

**Table 2:** OSDI changes during the trial.

Time	FAS analysis		PPS analysis	
	Moxibustion group	Artificial tear group	Moxibustion group	Artificial tear group
Baseline	41.32 ± 22.41	37.38 ± 18.79	42.74 ± 22.49	36.21 ± 19.45
Baseline—1 week	4.00 ± 13.46^△^	4.78 ± 17.47	4.62 ± 14.04^△^	5.86 ± 18.36
Baseline—2 week	9.83 ± 16.15^△^	6.34 ± 18.14^△^	10.58 ± 16.43^△^	7.23 ± 18.75^△^
Baseline—3 week	11.39 ± 17.15^△^	8.88 ± 18.28^△^	12.36 ± 17.46^△^	9.71 ± 18.81^△^
Baseline—4 week	12.71 ± 17.23^△^	8.12 ± 18.45^△^	14.06 ± 17.55^△^	9.00 ± 20.16^△^
End treatment-follow-up	−2.80 ± 16.29	4.47 ± 12.68	−2.80 ± 16.29	4.35 ± 12.96

*Note*: OSDI: ocular surface disease index; ^△^for *P* < 0.05, versus baseline within group; ^*∗*^for *P* < 0.05, versus artificial tear group at the same time point.

**Table 3:** VAS score changes during the trial.

Time	FAS analysis		PPS analysis	
	Moxibustion group	Artificial tear group	Moxibustion group	Artificial tear group
Baseline	64.78 ± 19.42	63.17 ± 20.28	66.05 ± 19.20	61.13 ± 20.44
Baseline—1 week	11.92 ± 17.11^△^	9.80 ± 17.03^△^	12.13 ± 17.49^△^	7.64 ± 15.13^△^
Baseline—2 week	19.55 ± 18.80^△★^	13.39 ± 19.62^△^	18.32 ± 19.18^△★^	11.83 ± 18.96^△^
Baseline—3 week	20.67 ± 18.92^△★^	14.68 ± 21.25^△^	19.60 ± 19.39^△^	13.65 ± 21.44^△^
Baseline—4 week	24.66 ± 20.82^△★^	15.51 ± 20.35^△^	24.27 ± 21.86^△★^	15.98 ± 19.85^△^
End treatment-Follow-up	−1.84 ± 20.44	2.81 ± 17.45	−1.84 ± 20.44	3.21 ± 18.10

*Note*: VAS: visual analogue scale.

**Table 4:** TBUT changes during the trial.

Time		FAS analysis		PPS analysis	
		Moxibustion group	Artificial tear group	Moxibustion group	Artificial tear group
Left eye	Baseline	2.89 ± 1.35	3.37 ± 1.74	3.02 ± 1.32	3.49 ± 1.75
	Baseline—2 week	−0.27 ± 1.67	−0.45 ± 2.20	−0.30 ± 1.77	−0.49 ± 2.34
	Baseline—4 week	−0.86 ± 1.90^△^	−0.56 ± 2.07	−0.84 ± 1.82^△^	−0.57 ± 2.12

Right eye	Baseline	3.02 ± 1.57	3.28 ± 1.70	3.12 ± 1.58	3.40 ± 1.71
	Baseline—2 week	−0.31 ± 2.08	−0.42 ± 2.22	−0.35 ± 2.20	−0.45 ± 2.36
	Baseline—4 week	−0.84 ± 2.08^△^	−0.82 ± 2.06^△^	−0.86 ± 2.12^△^	−0.84 ± 2.23^△^

*Note*: TBUT: tear film break-up time.

**Table 5:** CFS scores changes during the trial.

Time		FAS analysis		PPS analysis	
		Moxibustion group	Artificial tear group	Moxibustion group	Artificial tear group
Left eye	Baseline	1.97 ± 2.84	2.22 ± 2.96	1.86 ± 2.76	2.45 ± 3.06
	Baseline—2 week	−0.08 ± 2.28^★^	0.92 ± 2.37^△^	−0.09 ± 2.40^★^	1.09 ± 2.46^△^
	Baseline—4 week	0.25 ± 2.78	0.65 ± 2.90	0.28 ± 2.92	0.72 ± 3.12

Right eye	Baseline	1.51 ± 2.21	2.00 ± 2.60	1.41 ± 2.19	2.15 ± 2.68
	Baseline—2 week	−0.30 ± 2.09	0.47 ± 2.37	−0.33 ± 2.20	0.51 ± 2.50
	Baseline—4 week	0.22 ± 2.09	0.55 ± 2.60	0.25 ± 2.20	0.52 ± 2.81

*Note*: CFS: corneal fluorescein staining.

**Table 6:** Schirmer I test scores changes during the trial.

Time		FAS analysis		PPS analysis	
		Moxibustion group	Artificial tear group	Moxibustion group	Artificial tear group
Left eye	Baseline	9.05 ± 8.22	10.98 ± 9.05	8.42 ± 7.79	11.08 ± 9.30
	Baseline—2 week	−0.88 ± 8.21	1.30 ± 6.49	−0.86 ± 8.66	1.46 ± 6.92
	Baseline—4 week	−0.44 ± 6.69	0.44 ± 4.60	−0.70 ± 6.89	0.50 ± 5.00

Right eye	Baseline	8.64 ± 8.17	8.31 ± 8.22	8.05 ± 7.47	8.75 ± 8.28
	Baseline—2 week	0.02 ± 6.57	1.53 ± 6.18^△^	0.02 ± 6.97	1.82 ± 6.59^△^
	Baseline—4 week	1.41 ± 7.65	0.76 ± 7.03	1.23 ± 7.71	1.09 ± 7.72

## Data Availability

The datasets generated and analyzed during the current study are available from the corresponding author upon reasonable request.

## Abbreviations

CFS: Corneal fluorescein staining
DED: Dry eye disease
OSDI: Ocular surface disease index
VAS: Visual analogue scale
TBUT: Tear film break-up time
